# Secondary Malignant Tumors Arising in Nevus Sebaceus: Two Case Reports

**DOI:** 10.3390/diagnostics12061448

**Published:** 2022-06-13

**Authors:** Sohshi Morimura, Yasuhiko Tomita, Shinichi Ansai, Makoto Sugaya

**Affiliations:** 1 Department of Dermatology, International University of Health and Welfare, Narita 286-8520, Chiba, Japan; morimuras-der@iuhw.ac.jp; 2 Department of Pathology, International University of Health and Welfare, Narita 286-8520, Chiba, Japan; yasuhiko-tomita@iuhw.ac.jp; 3 Division of Dermatology and Dermatopathology, Nippon Medical School Musashi Kosugi Hospital, Kawasaki 211-8533, Kanagawa, Japan; shin8113@nms.ac.jp

**Keywords:** nevus sebaceus, malignant tumor, basal cell carcinoma, sebaceus carcinoma

## Abstract

Nevus sebaceus is a benign tumor that is present at birth and is often seen on the scalp or face. Secondary malignant tumors sometimes occur in nevus sebaceus in adulthood. Herein, we present two malignant tumors arose from nevus sebaceus. One is basal cell carcinoma on the face and the other is sebaceus carcinoma on the lower back, where nevus sebaceus rarely occurs. Basal cell carcinoma sometimes develops in sebaceus nevus after a few decades, seen usually on the scalp or face. Sebaceus carcinoma is a rare malignant tumor that arises in nevus sebaceus.

Nevus sebaceus is a benign hamartoma that is frequently seen on the scalp of infants at birth. Although nevus sebaceus is considered to be a benign tumor, secondary tumors including malignant tumors occur in it after a few decades. Nevus sebaceus usually manifests as a yellowish plaque or a nodule with verrucous appearance, which tends to appear along the Blaschko lines. Pathological features depend on stages. In the early stage, premature pilosebaceus cells are increased while epidermis shows almost no changes. In the following stage, mature pilosebaceus cells with abnormal apocrine glands develop and proliferation of epidermis starts. In the late stage, secondary tumors sometimes develop. Epithelial tumors such as trichoblastoma, syringocystadenoma papilliferum, and basal cell carcinoma sometimes occur secondarily.

In this paper, we show two cases of patients with malignant tumors including basal cell carcinoma and sebaceus carcinoma arising in the nevus sebaceus in adulthood.

A 48-year-old Japanese female was admitted to our department with a skin-colored plaque on the lower jaw (Figure 1A). Multiple black dots were seen on the upper part. Dermatoscopy demonstrated multiple maple-leaf structures. Her laboratory data were not remarkable. The whole plaque was removed by surgery. Histological examination revealed a peripheral palisade of basaloid cells with round nuclei with cleft formation between tumor cells and stroma (Figure 1B,C), which is one of the histopathological features to distinguish basal cell carcinoma from trichoblastoma. Proliferation of mature pilosebaceus tissues and ectopic eccrine glands in the dermis were detected with basaloid cell tumors (Figure 1B). Thus, we diagnosed the skin lesion as basal cell carcinoma generated in nevus sebaceus.

An 82-year-old Japanese female was admitted to our department complaining of bleeding from a red tumor covered with yellow granular papules, which was adjacent to a brown plaque on the right back (Figure 2A). A brown plaque had been present from birth, while it was not clear when the red tumor developed. The red tumor was removed surgically. Histology of the red tumor revealed that atypical basaloid cells form irregular lobular nodules with infiltrative growth pattern in the dermis (Figure 2B). Sebaceus differentiation with a foamy cytoplasm was present in the center of nodules (Figure 2C). The biopsy of brown plaque showed increased multiocular pilosebaceus glands and epidermal papilliform hyperplasia (Figure 2D). Therefore, we diagnosed this tumor as sebaceus carcinoma arising in nevus sebaceus. No extracutaneous metastatic lesions were detected by computed tomography.

Nevus sebaceus is a congenital hamartoma, which is clinically a yellowish plaque. Nevus sebaceus frequently occurs on the scalp and face. However, some cases with nevus sebaceus on the chest have been reported [1]. In total, 417 cases (92.6%) out of 450 cases were on the scalp and face, while 4 cases (0.8%) were on the trunk [2]. In one of our cases, nevus sebaceus was detected on the back, which is quite rare. We found only one case with basal cell carcinoma arising in the nevus sebaceus on the upper right back [3].

It has been reported that trichoblastoma is the most common secondary neoplasm that arises within nevus sebaceus [4]. Out of 243 cases with nevus sebaceus, only one case (0.4%) developed sebaceus carcinoma [4]. Another report demonstrated that 38 (8.5%) of 450 cases with nevus sebaceus developed secondary neoplasms, including syringocystadenoma papilliferum (2.7%), the most common tumor [2]. Basal cell carcinoma developed in 4 cases (0.9%) and was the most frequent malignant tumors in nevus sebaceus [2]. Nodular type of basal cell carcinoma arising from nevus sebaceus has been recently reported [5]. Sebaceus carcinoma occurred in only one case (0.2%) out of 450 cases of nevus sebaceus [2].

According to several articles, multiple tumors rarely happen in the nevus sebaceus simultaneously. Sebaceus carcinoma, trichoblastoma, and poroma were detected in one case with nevus sebaceus [6]. Coexistence of adenosquamous carcinoma, trichoblastoma, trichilemmoma, sebaceus adenoma, tumor of follicular infundibulum, and syringocystadenoma papilliferum was also reported [7]. The mechanism of multiple neoplasms happening in nevus sebaceus is still unclear, although diversity and different differentiation status of cells composing nevus sebaceus may be partially responsible for it.

In conclusion, we presented two cases of malignant tumors arising from nevus sebaceus. Nevus sebaceus should be removed in order to avoid malignant transformation. Skin biopsy is essential to avoid overlooking the disease.

## Figures and Tables

**Figure 1:**
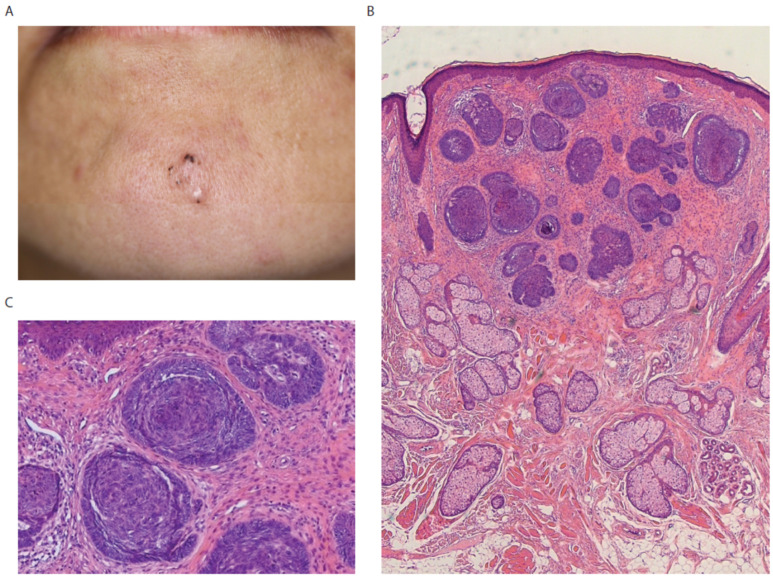
(**A**) A 10 mm skin-colored plaque slightly elevated with several black dots. (**B**) Multiple basaloid cell tumors in the dermis. Proliferation of mature pilosebaceus tissues and ectopic eccrine glands in the dermis. (**C**) Basaloid cell tumors show palisading pattern at the periphery with spaces between the tumor and the surrounding stroma. High magnification of (**B**).

**Figure 2:**
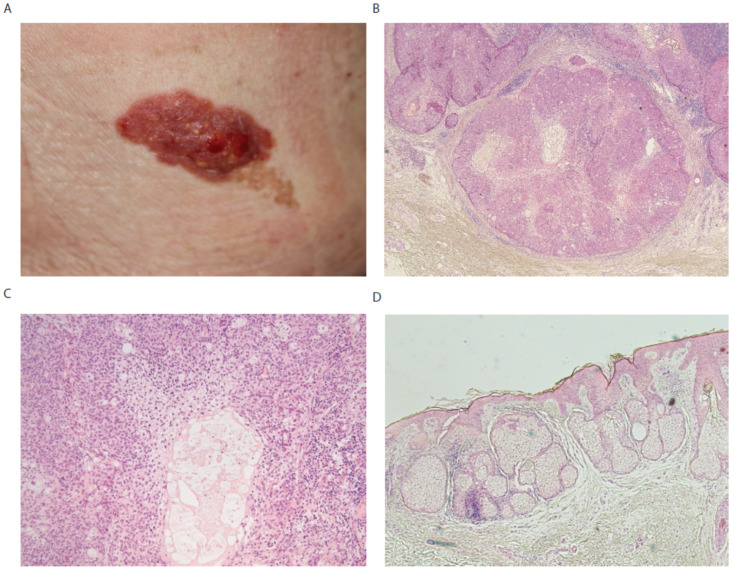
(**A**) A red tumor covered with granular papules adjacent to a brown plaque. (**B**) Atypical basaloid cells form irregular lobular nodules with infiltrative growth pattern in dermis. (**C**) Sebaceus differentiation with a foamy cytoplasm in the center of nodules. High magnification of (**B**). (**D**) Increased multiocular pilosebaceus glands in the dermis.
